# Lesions of the Blood

**Published:** 1835

**Authors:** 


### REVIEWS AND BIOGRAPHICAL NOTICES.

---

### VI.—Lesions of the Blood.

*A Treatise on Pathological Anatomy. By G. Andral, Professor to the Faculty of Medicine of Paris, Member of the Royal Academy of Medicine, etc. etc. Translated from the French by R. Townsend, M. D. and W. West, M. D. 2 vols. 8 vo. First American Edition, New York,* 1832.

(By a Correspondent.)

Although this work was reprinted nearly or quite as far back as three years ago, yet as the subject on which it treats is one that has hitherto attracted but little attention in this country, there is much reason to believe that it is not so generally known as it should be. It is not our design, at this late period, to enter upon a formal review of this valuable treatise; for, however ably this might be done, it would be but ill calculated to convey an accurate idea of the immense debt under which M. Andral has placed the physicians of the nineteenth century. It may be unhesitatingly asserted, that he has accomplished in pathological, what his countryman, Bichat, effected thirty-five years ago, in general anatomy; and, although he cannot be said, like his celebrated predecessor, to have created the science on which he so admirably and profoundly descants, it must be admitted that he has been the first to raise it to that exalted rank which it is destined, henceforth, to hold amongst the different departments of the healing art. Like the author of the "Anatomie Generale," the name of Andral will be handed down to the latest posterity; and his work be acknowledged as an imperishable monument of his untiring industry, his genius, and his talents.

Pathological anatomy may justly be considered as the creation of the present century, for it is only within the last twenty or thirty years, that it has begun to assume the form of a regular science. The works of Bonetus, Wepfer, Valsalva, Schenke, Fantoni, Vander-Wyl, Morgagni, and others, although abounding in valuable facts, contain little more than mere collections of cases, drawn up frequently with so much diffuseness and so great a want of accuracy, as to render them, in many instances, of comparatively little utility. The pathological anatomy of these writers, indeed, is quite a different thing from the pathological anatomy of the present day; for, as they were in complete ignorance in regard to the properties and functions of the various tissues which enter into the composition of our frame, it follows, as a matter of course, that they must have been utterly incompetent to detect those minute alterations which are consequent on disease, and which have since been so ably described by some of the French and German writers. The publication of the "Anatomie Generale" may, therefore, be regarded as the period from which we may date the history of modern morbid anatomy; the brief but lucid pathological remarks which the author of that great work has appended to his account of the normal tissues, form unquestionably the foundation of all that has since been done or written on the subject; and had he been permitted by an all-wise Providence to reach the age which is ordinarily allotted to man, there is little doubt but that he would have made the study of morbid anatomy his chief pursuit. As it was, his last days were devoted to the subject, and the little treatise which was published a few years ago, under the title of the "Last Course of Xavier Bichat," although wofully mangled and distorted by the American translator, fully attests the growing interest which that great master-genius manifested in the cultivation of this delightful department of medicine.

At no period has the study of morbid anatomy been pursued with greater ardor and success than within the last fifteen years. During this short space, a vast body of facts has been accumulated, and scarcely a day has elapsed without some interesting discovery. The march is still onward, and it scarcely requires the aid of prophetic vision to foresee the immense benefits which a proper and profound cultivation of this science is destined to confer upon the human race, by the changes and improvements which it must necessarily effect in the treatment of accidents and diseases. Whilst mere descriptive anatomy will become dry and wearisome, or be tolerable only as it may have a tendency to lead to a more accurate comprehension of the various functions of the body, or to a more precise knowledge of the relations which one organ or structure bears to another, pathological anatomy, dressed in the inviting garb of morbid physiology, will enlist the admiring attention of the student, and draw him, as with an irresistible charm, to her shrine.

Nowhere, perhaps, is this science more fully appreciated at this moment than in the French and German schools, in which it is now uniformly taught as essential to graduation. So fully was the celebrated Dupuytren, whose death has been so recently deplored by the medical profession throughout the whole world, impressed with the importance of pathological anatomy, that he made a provision of $40,000, in his last will, for the purpose of instituting a chair for that department; thus compensating, if I may be allowed the expression, in some degree, for his own loss, by the new impulse which these means will have a tendency to excite amongst the younger practitioners of medicine in the French capital. In England, too, the science is making rapid strides; and the creation of separate professorships in her more respectable institutions, and the writings of some of her more distinguished physicians, especially those of Abercrombie, Craigie, and Carswell, all attest, incontestably, that this branch of anatomy will hereafter receive that attention, to which, from its vast importance, it is so justly entitled.

But, whilst we thus look with delight upon the cultivators of this science amongst other nations, is it not a subject of deep regret and mortification, that America, already so distinguished for her intellectual strength and her improvements in some of the other departments of science, should not be able to afford a single individual eminent for his attainments in pathological anatomy? With the exception of Horner, Geddings, Morton, and one or two others, there is scarcely a physician that has manifested the slightest interest in behalf of this science; and to show how little it is regarded by the profession generally, it is only necessary to refer to the fact, that there is not at this moment a *single school* in which it is taught as a regular course of lectures. This, indeed, is a most melancholy circumstance, and it were high time, it seems to us, both for the honor of our profession, and for the well-being of our fellow men, that this unwholesome lethargy should be converted into something like a healthful reaction. The time is at hand when our whole system of education must be reformed, and when the student must begin to learn his profession by commencing at the Alpha instead of the Omega, as has been heretofore the case.

But to return to the work of M. Andral. As already hinted, it is not our design to enter into an elaborate account of it, but merely to give such extracts, interspersed with occasional remarks, as will be likely, from their novel character, to interest the reader. With this view we shall take up the consideration of the pathology ofthe blood, which occupies the last thirty pages of the first volume of the work before us.

There was a period in the history of medicine, when the blood was invested with a degree of importance, of which, in more modern times, it has been in a great measure deprived. For many centuries almost every disease was attributed to a morbid condition of this fluid, whilst the solids which are formed by it, were entirely neglected as objects of little or no consequence. Though it cannot be doubted that during the prevalence of the humoral pathology, as it has not been unaptly denominated, too much stress was placed upon the blood, yet it is equally true that during the reign of solidism, which immediately succeeded, and which, until the present moment, has been the prominent doctrine of physicians, this fluid has lost too much of its former consequence. "The humoral pathology," says Bichat, "has no doubt been carried too far; but it is, nevertheless, founded on truth, and *in a great many cases*, it must be confessed that almost every thing should be referred to a deranged state of the humors." The idea which was thus thrown out by Bichat was almost forgotten, as it was neither followed up by himself nor his contemporaries; but in the progress of science it is easy to foresee, to use the language of M. Andral, that the theory of exclusive solidism must be eventually rejected, as being incapable of affording an explanation of every case of disease; and that it will be necessary, ere long, to have recourse to some other system for a more satisfactory solution of the difficulty.

Although I do not feel disposed to attach that great importance to the blood which the advocates of the humoral pathology did, it cannot be denied, that it is decidedly the most essential fluid in the animal machine, inasmuch as it furnishes the various materials which dispense vitality and nourishment to the different tissues of the body, as well as vigor to its several organs, serving thus, in the expressive language of some of the ancient writers, as the *pabulum* of life. From it, all the solids are formed, and all the other fluids secreted; and hence it may be justly considered as the basis of every part of the body, as without it, it would be utterly impossible for any growth whatsoever to take place. Pervading every portion of our frame and penetrating every fibre, however minute or however constituted; acquiring constantly new properties as it moves through the lungs, and losing them again in its meanderings through the body; it is not at all improbable, that, whilst it thus fertilizes the various textures and organs, it may convey alike the elements of general health and of general disease. As long as it retains its normal condition, it must give rise to normal actions, calculated to stimulate the whole system, and rouse it to the performance of its natural functions. An alteration in its natural properties, although so slight as to be scarcely cognizable to our senses, would be followed, it is reasonable to suppose, by a corresponding impression upon the solids. In inflammation, especially in that variety called pleurisy, it undergoes important changes, assuming, as we shall presently see, a peculiar appearance, termed the buffy coat; in dropsy it has a thin watery aspect, not unlike weak claret; in plethora, on the contrary, it is thick, of a deep red, firm, and opake.

The blood, then, being in such intimate combination with the solids, it would almost follow, as a necessary consequence, that they would exert a mutual influence upon each other in disease, especially if it produced considerable general derangement. "If we analyze the blood and the solids," says M. Andral, "we discover that they are made up of the same proximate principles. If we examine their physical structure, we find it identical, both consisting of globules mixed with an amorphous substance." Bordeu acknowledged this identity of composition when he said that the blood is fluid flesh, "*Le sang est de la chair coulante.*"

"Thus then in the three-fold respect of the vital phenomena,* intimate structure, and chemical composition, no line of demarcation can be drawn with strictness and precision between the blood and the solids. Physiologically speaking, it is impossible to conceive that one of these two parts of the same whole could be modified without the other being so likewise. On the one hand, inasmuch as the blood nourishes the solids, and as without its presence they cannot

---

*It is admitted that the phenomena called *vital* do not display themselves unless in certain states of arrangement of the particles of the body, termed *organization;* but the meaning of this last word is far from being fully determined, and we must not imagine that manifestations of vitality can occur only where there exists an organization such as is observed in the higher order of animals, and such as we are accustomed to figure to ourselves as existing in every case. If we follow the series of living beings, we shall find the vital organs gradually diminishing in number and complexity; nay, even disappearing altogether, and yet life continuing. Life exists likewise in the seed in vegetables, and in the drop of fluid that constitutes the first rudiments of the embryo in animals; yet in these, we find still less appearance of what is called *organization*, than there is in the blood. We see, then, that vital actions take place without the presence of that peculiar formation to which common opinion attaches the idea of vitality; so that observation, far from leading us to infer that certain conditions of arrangement are necessary for the manifestation of life, teaches us that in a thousand different cases life is revealed to us, not by forms but by acts.

support life, the state of the solids cannot but be influenced by the state of the blood. The chemist might as well say that the nature of a body does not depend on the nature of the elements that compose it. On the other hand, the solids, considered with respect to their relations to the blood, form but two classes: the one contributing to *make* the blood, such as those concerned in the actions of absorption, digestion, arterial circulation, and respiration; the other contributing to *unmake* it, those, namely, concerned in the actions of venous circulation, secretion, and nutrition. No one solid, therefore, can undergo the slightest modification, without producing some derangement in the nature or quantity of the materials destined to form the blood, or to be separated from it. Physiology, then, leads us to the conclusion that every alteration of the solids must be succeeded by an alteration of the blood, just as every modification of the blood must be succeeded by a modification of the solids. Viewed in this light, there is no longer any meaning in the disputes between the solidists and the humorists; the system appears to constitute but one great whole, invisible in the state of health as well as in that of disease; the division of the parts of the body into solids and fluids seems to be a distinction of small importance, and one that is not always just, since it ceases to exist in the intimate structure of the organs, in which all the grand vital phenomena take place, and in which also occur all the changes that constitute the morbid state." p. 390.

Another proof of the intimate union subsisting between the blood and the solids, may be derived from the well-known fact, that the elements of most of the organs, and even some of the secreted fluids are to be found in it. These elements are probably held in solution in a certain quantity of water, and as the blood moves through the various textures of the body, it no doubt constantly deposits them in such a manner as to contribute, on the one hand, to the nourishment of the system, and, on the other, to the repair of its several parts, so as to enable them to execute their healthy functions. Thus, as it passes through the osseous texture, it deposits phosphate of lime; through the muscular texture, fibrin; and so on with the rest of the tissues; whilst in the organs it deposits, though probably in a more complicated manner, the materials which constitute the recrementitial, as well as some of the excrementitial fluids.

When blood is drawn from a vein or an artery, observation has long since made us familiar with the fact, that it separates, in a very short time, into two parts, the one of them being solid, the other fluid. The former, generally termed the crassamentum, cruor or clot, is principally composed of fibrin and coloring matter; the other, on the contrary, called the serum, consists almost entirely of water and albumen, the latter ingredient being the sole cause of its coagulating when exposed to heat, alcohol, or the dilute mineral acids. The relative proportion which these two parts bear to each other is an interesting object of inquiry, and must, if possible, be accurately determined before we shall be able to speak satisfactorily in relation to their increase and diminution caused by disease. In the healthy condition of the system the quantity of serum has been usually estimated to be a little higher than that of the crassamentum; but according to Dr. Scudamore, the mean average of twelve experiments gave nearly fifty-six and a half per cent of cruor. My own observations, however, would lead me to an opposite conclusion; for, in the great majority of my experiments on this subject, the quantity of serum most decidedly preponderated over that of the crassamentum. The proportion of this fluid, as must be familiar to every physician, is always greater in persons of a debilitated habit of body, than in those who are strong, healthy, and vigorous; it is greater again when coagulation takes place under a low degree of temperature; or in those who are impoverished by poor living or long abstinence. The observations of M. Andral on this point are interesting, and deserve to be noted. "In a great many morbid states the blood," says he, "during life or after death, presents different appearances, constituting so many pathological conditions."

"Thus the fibrin may be altered either in quantity or in quality. In the first place, there are cases in which this principle is more abundant than usual, or at least in greater proportion relatively to the water and albumen. In such cases the blood, when drawn from a vein, forms in the vessel that receives it a clot with little or no serum. These are however to be divided into two classes. In the first class of cases, the fibrin constituting the clot, still contains a pretty large quantity of serum, which may be separated from it by pressure; in these the coagulum has but little density. In the second, on the contrary, the clot is very dense, and a little fluid albumen can with difficulty be squeezed out of it. In the first class the relative increase of quantity of the fibrin is only apparent; in the second, it is real. We must take care not to confound them, as they belong to different states of the system. The very fibrinous blood is commonly called *rich blood:* it may either depend simply on a vigorous constitution, or on certain morbid states.

"In place of being superabundant in the blood, the fibrin may, on the contrary, be quite the reverse. There are, in fact, some persons whose blood, when taken from a vein, presents but a very small coagulum in proportion to the large quantity of serum in which it appears. But, here likewise we must make a distinction. The diminution of quantity of the fibrin may be only apparent; as happens when its particles being very strongly condensed, are much closer to each other than in their natural condition; the clot is then very small and remarkably firm; this occurs very often, for instance, in patients suffering under acute rheumatism. In other cases, the clot is not only very small, but also very soft; and there is then really a deficiency of fibrin. This may be observed in many cases of chronic disease, or in persons with a slender muscular system, and habitually pale skin." p. 392.

The changes produced in the composition of the blood by repeated bleedings, are of considerable interest in a practical point of view; but as yet our knowledge on this subject is very slender. Very recently Dr. Andrews, of Europe, instituted some experiments on this point, in the hope that he might be able to throw some light on it. For this purpose, he made choice of the blood of the calf. The animal was bled from a large orifice in the jugular vein, till symptoms of syncope appeared, and the operation was generally repeated at intervals of twenty-four hours, during which it was usually once fed upon a mixture of meal and water. The appearance of the blood thus drawn became greatly altered by the successive abstractions; in the first bleeding the crassamentum was very large, and a portion of the red globules remained unattached to it, but as the operation was repeated, it gradually diminished in bulk, whilst its consistency augmented, till upon the fourth bleeding, when it appeared like a small contracted ball, immersed in a large quantity of serum.*

This experiment was a number of times repeated, upon different animals, and invariably with the same results; whence it may be fairly inferred that a diminution of the crassamentum, with a corresponding increase of serum, is a very constant, if not an invariable effect, of the repeated abstraction of this fluid from the system. Dr. Andrews further ascertained that there is a sensible decrease of the albumen and

*North American Archives of Medical Science, No. viii. p. 135.

salts at each bleeding; he states, however, that the diminution is very variable, and that even after the fourth bleeding it scarcely amounts to one and a half per cent. In the globules the same diminution takes place, being not unfrequently reduced to less than one-half of their original quantity. These experiments are unquestionably of a highly interesting nature, and it is to be hoped that some physician, endowed with industry and talents, will soon be induced to repeat them. The results would at least be curious, if not calculated to lead to useful precepts in practice.

"The force that tends to keep the globules of fibrin at a certain distance from each other during life, may be so modified as that they shall have a tendency to run together, as they naturally do after death; and hence may result the spontaneous coagulation of the blood in its vessels during life. There have been too many cases of this nature observed, for us of the present day to attempt to deny the possibility of its occurrence. Sometimes it takes place without any known cause; and sometimes it appears to accompany a state of irritation in the parietes of the containing vessel. When the blood once becomes solid, it displays indubitable symptoms of vitality; vessels are produced, and secretions formed in it; and different alterations of nutrition, resembling those observed in the tissues, may also occur. If we examine whence this coagulated blood derives its vitality, we find that it cannot partake of the common life of the rest of the body, since it very often merely touches the surrounding tissues, without being in any manner continuous with them. We must therefore admit that these polypiform concretions, or *polypi*, as they are called, may possess a proper vitality, by means of organs they have created themselves." p. 393.

Many cases of sudden death have the singular effect of impeding the coagulation of the blood, or in entirely preventing it. This is strikingly exemplified in persons who are suddenly destroyed by lightning and electricity; a violent blow on the stomach, or severe injury of the brain; by the bite of venomous serpents, as the rattle-snake and copper-head; by acid vegetable poisons, such as laurel-water or prussic acid; excessive fatigue, as in hunting down wild animals; and even violent agitation of the mind.

It is extremely probable that some of the causes here enumerated, do not invariably produce the effects that have been attributed to them. We are informed, for instance, by Gumilla, an old Spanish missionary amongst the South American Indians, that he examined several animals that had been killed by poisoned arrows, in which the blood was completely inspissated, although death took place almost instantaneously: and in regard to death from lightning, the testimony of Sir Charles Scudamore is precisely of the same import. He found that blood through which electric discharges were transmitted, coagulated as quickly as that which was not electrified; and in small animals, destroyed by the discharges of a powerful galvanic battery, the blood in the veins was always in a solid state.

"In certain cases in which the natural consistence of the fibrin is increased, or even without any very decided augmentation of its density, another very remarkable phenomenon is observed; the portion of fibrin at top of the coagulum parts with the coloring matter, and forms a whitish, yellowish, or slightly greenish layer, that may vary in thickness from less than a line to some inches. This layer is known by the name of the *buff*, or buffy coat; and although its formation may be more or less favored by certain circumstances that have nothing to do with the accompanying morbid state, such as the size of the orifice, the manner in which the blood flows, and the form of the vessel that receives it, yet it is not the less true that it can occur only in certain states of the system which I shall presently explain. The buffy coat is formed of pure fibrin, with which is mixed a certain quantity of serum, which, according to the researches of Dowler and of Gendrin, contains much more albumen than the serum of the rest of the blood. The greatest analogy exists, both in respect of appearance and of chemical composition, between the buffy coat of the blood and the substance that constitutes the false membranes of the serous cavities." p. 395.

The inflammatory buff, or, as it has not been unfrequently termed, the *pleuritic crust*, begins to form as soon as the blood commences coagulating, appearing at first like a thin viscid layer, of a light reddish tint, which is spread either uniformly over the whole surface of the fluid, or occurs in small insulated spots, looking like so many little islands in the midst of a body of water. This viscid substance may be drawn out in the form of shreds or filaments, which, on becoming firm, assume a white reddish aspect. The coagulation of inflamed blood begins much sooner than that of healthy blood, and is also more speedily completed. The buffy coat forms as soon as the crassamentum contracts sufficiently to detach itself from the walls of the vessel in which the fluid has been received, but it does not acquire its full density until the coagulating process is perfected. When this has taken place, it will be found to be dense, elastic, slightly diaphanous, strongly adherent to the cruor which it covers, smooth on its free surface, and rough on the other. If it be carefully separated from the crassamentum, washed in cold water, and then immersed in alcohol, its elastic properties will be greatly augmented, and the membrane will present the appearance precisely of a half tanned hide, or of the proper uterine tissue during pregnancy, making thus a beautiful preparation.

As long as it remains soft the texture of the buffy coat may be completely dissolved in tepid water; but if it has once become concrete, this can be only imperfectly effected. On adding a decoction of nut-galls, the solution thus obtained yields a yellowish coagulable matter; whilst the insoluble part, on being macerated six or eight hours in water, becomes friable, white, rough, and as it were pulverulent, greatly softened and distended, and forming about the twenty-fifth part of the whole mass of the buffy crust. Dilute acetic acid and a saturated solution of the nitrate or carbonate of potass, also possess the property of dissolving this substance, which is augmented in density and consistence by the mineral oxides, in a concentrated state. These chemical characters, however, are, it would seem, by no means constant or uniform; for Galber asserts, that he has frequently seen buffy coats that were neither soluble in pure tepid water, nor in a solution of the nitrate of potass.*

The immediate cause of the buffy appearance of the crassamentum is sufficiently obvious; the globules or other matter which give it the red color, begin to subside before the coagulation is completed, whereby the upper part of the clot is left without them. The remote cause of this occurrence is by no means so obvious as the proximate, notwithstanding the many

*Gendrin's Histoire Anatomique des Inflams. t. ii. p. 431.

experiments that have been made to discover it. Hewson thought that the fibrin became specifically lighter, and of course the red globules relatively heavier, whence they would be disposed to sink to the lower part of the clot; he also thought, which, however, has since been proved to be unfounded, that the blood coagulated more slowly. John Hunter endeavored to account for the appearance by the firmer coagulation of the fibrin squeezing out, as it were, the red particles; but this would scarcely, as observed by Dr. Bostock, explain why the upper part of the crassamentum was left without them. By the celebrated Hey, of Leeds, it was referred to a preternatural action of the vessels, in consequence of which the different constituents of the blood were more intimately blended together. Neither of these opinions, however, it seems to us, are capable of affording a satisfactory solution of the difficulty; and on the whole, the doctrine of Dowler, promulgated for the first time in 1822, is probably more correct than any other that has yet been proposed. This is founded on the well established fact, that inflamed blood always contains a very large proportion of serum, which, by diminishing its vicidity, readily allows of the subsidence of the red globules.

The formation of the buffy coat is also materially influenced by the size of the orifice of the vein, and the dimensions and shape of the vessel in which the blood is received. Sydenham long ago asserted that the inflammatory crust never makes its appearance when this fluid is permitted to flow *guttatim*, or drop by drop; and De Haen was well acquainted with the fact, that a deep, narrow vessel is much more favorable to its formation than one of opposite dimensions. The observations of these distinguished physicians, have been recently confirmed by the results of the experiments of Belhomme, Gendrin, and other pathologists, and may therefore be regarded as fully entitled to our confidence. Time will not allow us to enter into any further remarks on this interesting topic; the facts which have been here referred to are of considerable practical importance, and as such, if for no other purpose, they deserve to be remembered in making up our minds as to the propriety of the repeated abstraction of blood, when it presents the buffy coat.

"The albumen, which always exists in a small quantity in the coagulum, and which, united with water, almost exclusively forms the serum, may be modified as well as the fibrin. In the first place, there are cases in which, in a given quantity of serum, the albumen is found in much greater quantity than usual in proportion to the water; of this we may easily be convinced by exposing the serum to heat. The researches of Dr. Traill, which are confirmed by the more recent ones of M. Gendrin, have proved, that in the state termed inflammatory, the serum of the blood contains nearly twice as much albumen as in the healthy state. This increase of the albumen may be detected simply by the touch, the serum being then remarkably viscid. In other cases, on the contrary, the very small coagulum obtained by heating the serum, the greatest part of which evaporates, proves how much the quantity of albumen is diminished.

Is it to a particular alteration in the nature of the albumen of the blood we are to attribute the presence of a *mucous layer* that has been sometimes observed by M. Gendrin at the bottom of the serum, or suspended in it like a cloud? In one of the cases he mentions, the blood was taken from an individual affected with empyema; in another, there was a vast abscess in one of the thighs.

The serum, in respect to its composition, presents several varieties that we must not confound: 1. it may contain at the same time much water and much albumen; 2. the latter principle may predominate, the quantity of water remaining the same, or even being diminished; 3. the reverse may take place, and the serum be composed of much water, and little albumen.

Whatever be the composition of the serum, it is sometimes found in small quantity in proportion to the coagulum, and sometimes the reverse is the case. All these differences should be noted, as corresponding to so many particular morbid states." p. 395.

During derangements of health, the serum, like the crassamentum, exhibits various peculiarities. Thus, in inflammation, the serum is more viscid, and of a lighter color than usual, containing, in fact, scarcely the slightest trace of coloring matter. These appearances are less evident when the clot does not float in, or occupy the centre of the serum. This extraordinary viscidity is owing to the fact already alluded to, that the fluid in question contains almost a double proportion of albumen in inflammatory affections than it does in health.

In some instances the serum presents the appearance of whey, with white streaks floating upon its surface: in others, it has been found to have oil in it; and in others, again, it has been known to be as white as milk, the clot retaining its usual aspect. Mr. Hewson, who considered the red particles of the blood to be flattened, attributed the color of the serum, in these cases, to the presence of very minute globules. Mr. Hunter, in his work on the blood, states, that he once saw the serum separate from, and swim upon the uncoagulated fibrin, immediately after the fluid had been abstracted from the arm; and he likewise mentions that in jaundiced persons the serum, although not bitter is of a deeper yellow than ordinary; a fact which has since been abundantly verified by other observers.*

The presence of oil in the serum of the blood is probably of more frequent occurrence than has been generally imagined. The cases of it upon record, however, are probably few, and the only ones with which I am familiar are those narrated by Dr. Traill, of Liverpool.† In the last case which he has reported, the serum was of a light straw color; and when recent, had the appearance of a homogeneous fluid, of the consistence of cream; but on being kept for a short time it coagulated so firmly as to be poured with difficulty from a glass bottle. On analysis it yielded a large proportion of albumen and *oil*, of a character so pure as almost to burn when exposed to the flame of a lamp. In all the cases that have fallen under the notice of Dr. Traill, the patients, he observes, have labored under internal inflammation, affecting the chylopoietic viscera, and have been more or less addicted to the abuse of strong liquors.

In a case which came under the observation of the writer of this article, in 1832, the surface of the blood, which had been received into a small tin-basin, presented a white cream-like aspect, having formed a layer of about the eighth of an inch in thickness. The patient was a young man, thirty years of age, of intemperate habits, and complaining at the time he was

*Mayo's Outlines of Human Physiology, p. 32.

†Edinburgh Medical and Surgical Journal, vol. xxvii.

bled, of considerable pain in his head and chest, the symptoms in the latter part having been of a pleuritic nature. The blood, as it flowed from the arm, had a perfectly healthy appearance, but it had scarcely stood ten minutes before it assumed the aspect above mentioned. Some of this fluid, having been transferred into another vessel, was allowed to remain at rest until the next morning. On examining it, it was found slightly coagulated, of a strong saline taste, and somewhat heavier than water. It readily coagulated on the addition of alcohol, heat, and the mineral acids; and on being inspected with a microscope it exhibited no appearance whatever of globules; whence the inference is conclusive that it was composed essentially of albumen. The cruor, in this case, was somewhat larger than usual, and of course the quantity of serum less. On repeating the bleeding twenty-four hours afterwards, the blood presented precisely the same appearance.

The blood, considered as a mass, may be altered or affected in various ways. All these changes should be noted, as they correspond to so many particular morbid conditions of the system. In another part of this treatise, M. Andral has mentioned cases in which the blood contained not only the different elements of the secreted fluids, but also various other morbid productions, such as pus, encephaloid matter, entozoa, and calculous concretions. In whatever way these substances gain admission or are developed in the vessels, there is no doubt that by combining with the blood, they may so alter it as completely to change its physical properties, and thus vitiate the living current.

"Now, who could assert that in such cases the blood is not deeply altered in its nature. Besides, most commonly there are at the same time in the texture of many of the solids, morbid secretions, purulent or otherwise, formed of a matter that has the greatest analogy to that found in the vessels. Amongst the cases of this description I have had an opportunity of observing, I shall adduce the following. In a woman who died at *La Charitie* with all the symptoms of a chronic affection of the lungs and of the digestive passages, I found in front of the vertebral column an enormous tumor composed of an agglomeration of lymphatic ganglions, which, instead of their natural tissue, presented merely an inorganic pap, of a greyish or reddish color. A similar substance appeared in the liver, in the form of roundish isolated masses; it was also found in the spleen, in the cells of which it appeared to be deposited in place of the blood they usually contain; lastly, in several points of the lungs, the lobules were infilterated with the same substance. But that was not all; for in both lungs a great number of the branches of the pulmonary artery contained, instead of blood, a curdy matter of a reddish grey color, resembling in appearance the morbid matter found in the mesenteric ganglions, liver, spleen, and lungs. The right cavity of the heart, the pulmonary artery, and its first divisions, contained a blood of little color, and poor consistence. In another woman, who had a broken down cancer of the uterus, all the veins of that organ, and the trunk of the vena cava up to its passage under the liver, were full of a semifluid sanious matter of a greyish or reddish white. In a man far from being advanced in life, who had in a great many organs, encephaloid masses in a state of softening, the inferior vena cava, the renal and splenic, and some branches of the superior hepatic vein and of the pulmonary vessels, were filled with a kind of detritus of a reddish grey color, without its adhering to the venous parietes, which, in this case, as well as in the preceding cases, presented no appreciable trace of alteration. Facts similar to those just mentioned have also been observed by others. Thus, Beclard mentions a case in which the heart and principal trunks of the vessels were filled with a solid clot, the interior of which presented numerous collections of encephaloid matter. M. Velpeau found a mass of encephaloid in the midst of a clot of blood contained in the vena cava. He also cites a case of a man that died almost suddenly, after having shown some symptoms of cerebral congestion, and in whom, upon examination, there was found through the whole extent of the circulatory system, a blood of a pultaceous consistence and blackish red color, resembling the matter of certain abscesses of the liver." p. 397.

The alterations of this fluid, as observed by M. Andral, may be ascertained in still another way than by mere inspection or chemical analysis. It is well known that the blood of one animal may be introduced into the veins of another, of the same species, without causing any particular injury. Where this, however, is not the case, and where the blood of a diseased individual, when transferred into the vessels of another, acts as a poison, the conclusion forces itself irresistably on the mind that the nature of this fluid is really changed. The following remarks, which place this subject in a very clear light, are worthy of special notice.

"M. Gendrin, in his work on fevers (vol. ii. p. 145) gives an account of a flayer whom he attended in a putrid fever with an eruption of gangrenous pustules. An ounce of blood drawn from one of the patient's veins was injected into the cellular tissue of the groin of a cat. The consequences to the poor animal were copious vomitings of bile, at first yellow, and then greenish, dyspnœa, a small, frequent, and irregular pulse, a dry and brown tongue, a constantly increasing prostration of strength, and, towards the close of the scene, some slight convulsive motions at intervals. Death ensued in six hours and fifty minutes after the injection. The appearances observed on examining the body are described as follows: — The skin of the groin did not adhere to the subjacent parts; the cellular tissue was soft and almost pulpy, and of an ashy yellow color; it exhaled a fetid odor, and was dotted with small red spots; the gastromucous membrane of the stomach and intestines was in the natural state, that of the respiratory passages was of a reddish brown; the lungs, especially the left, contained black blood, and were full of brownish black spots; the blood throughout the whole body was black and fluid; in the left pleura were about two ounces of very serous black blood; the heart was soft and flaccid; there was no appearance of lesion in the brain or spinal marrow; the body speedily began to exhale a fetid odor.

Some blood that proceeded from an epistaxis that occurred in the same patient, was injected into the femoral vein of a dog. The animal exhibited the same series of symptoms as the one in the preceding experiment, which, in like manner, terminated in death.

In another work (*Histoire des Inflammations*, vol. ii.) M. Gendrin relates some experiments in which he injected into the veins of animals the blood of persons laboring under confluent small-pox. Very severe symptoms, which rapidly proved fatal, ensued; and on opening the bodies, several organs were found in a state of high inflammation.

MM. Dupuy and Leuret introduced into the cellular tissue and veins of a sound horse, blood that came from horses affected with malignant anthrax, ("*charbon*") and thus succeeded in producing the disease. It is, then, beyond all doubt, that in this case the blood itself was altered in its nature, since it proved capable of transmitting the affection.

These facts bring to our recollection some others, for an account of which we are indebted to the celebrated Duhamel. He has related a case where an ox, that was over-driven, having been slaughtered at an inn at Pithivers, the butcher put into his mouth, for a few moments, the knife he had employed for the purpose. The consequence was, that in some hours afterwards his tongue swelled, his breathing became difficult, and then blackish pustules broke out all over his body: at the end of four days he died. The inn-keeper wounded himself with a bone of the same ox in the palm of his hand; his arm mortified, and he died in seven days. Two women having received some drops of the blood of the same animal, the one on her hand, the other on her cheek, these parts were seized with a gangrenous inflammation. Is it not likewise the simple contact of the blood of diseased animals that produces malignant postule in man?

From these facts we must conclude that, under certain circumstances, the blood may be altered in its intimate nature, so as to acquire noxious properties, which display themselves when it is mixed with the blood of healthy animals." p. 399.

In cholera, and many other diseases, the blood, as is well known to every accurate observer, is very materially altered, but whether primarily or consecutively it is not very easy to determine. In the former of these disorders, the fluid is drained of its serum; it assumes a dark, semi-concrete appearance; and coagulates almost the moment it is drawn from the vessels, owing probably to the loss of a certain quantity of nervous influence. The effects of cutting the pneumogastric nerves on the blood were long ago pointed out by Dupuytren, and have been more recently elucidated by the experiments of Professor Meyer of Germany. This physiologist found that, whenever both pneumo-gastric nerves are tied in animals, the blood in the whole pulmonary system coagulates and the coloring matter separates from the fibrin. Professor Dupuy, of the veterinary school at Alfort, further ascertained, as he thought, in repeating these experiments, that the blood loses a considerable quantity of fibrin; for instance, he took a certain quantity of this fluid from the carotid artery, and found that it contained twenty-one grains of fibrin. In four hours after, the same quantity contained only nineteen grains; at the end of sixteen hours, eighteen grains; at the end of forty hours, twelve grains. In fifty-two hours after the operation the animal died in a state of suffocation; when the same quantity of blood, still taken from the carotid, contained but seven grains of fibrin. The question here naturally presents itself, was this progressive diminution in the quantity of fibrin produced directly by the division of the nerves; or was it merely indirectly, by disturbing more and more the progress of sanguification in the lungs? In all probability the section of the nerves had nothing to do with it; for it is not at all unlikely, though this is not mentioned, that the animal, at each bleeding, lost much more blood than was necessary for the purposes of the experiment; and, if this be admitted, the facts observed by M. Dupuy, although he attempts to account for them in a very different way, will be found to accord precisely with the results of the experiments of Dr. Andrews, alluded to in a previous part of this paper.

"I have now stated the facts and arguments which, in the present state of the science, ought to lead us to acknowledge the existence of certain alterations in the blood. What I have already said on the subject, in my opinion, sufficiently demonstrates not only that these alterations are real, but also that they are often primary, that they precede those of the solids, and that, consequently, the origin of many diseases lies in the blood. If it is true that the mass of the blood may, in certain cases, be primarily altered, it follows that the existence of general disease is not merely imaginary. In fact, when all the tissues thus receive a vitiated blood, is it not consistent with sound physiology to admit that their regular modes of vitality, nutrition, and secretion, must be more or less deeply modified. We must either admit this conclusion, or deny the influence which, according to every physiologist, the blood exerts over each solid. It may then happen that one or more organs are affected in a more decided manner than the rest, and there may thus be produced in them various lesions that are only accidental and secondary; but it is not in these lesions the origin of the affection lay; it is not on them all the symptoms depend; nor, lastly, is it to them alone we are to have recourse to throw a light upon the true nature of the disease, as well as upon the treatment proper to be pursued. Experience also teaches us, that these lesions may be either severe or slight, present or absent, similar or dissimilar, and that, notwithstanding, the disease, though presenting so many different shades in its variable symptoms depending on these lesions, does not the less exist, as it really consists in the constant symptoms depending on the state of the blood.

We must not, however, forget, that different alterations of the blood may exist, and have actually been observed, in persons presenting every appearance of good health. But such persons are on the brink of disease, and if any cause whatever chances to derange the equilibrium of their system, some of the morbid phenomena that occur are sure to depend on the state of their blood."

We have thus passed in review some of the more interesting facts recorded by the author of the "Anatomie Pathologique," and have added such remarks as seemed to be relevant, in further explanation, to the various topics which he has discussed in his able treatise. From the numerous facts that have been adduced, it may be justly concluded, we think, that the blood may not only become a frequent vehicle of disease, but that in many instances it is affected long before there is any manifest derangement of the solids. From the large surface upon which the chyliferous vessels open in the intestinal canal, it is not at all improbable that the elements of disease may, more or less readily, find their way into the current of the circulation, and thus vitiate the solids as they move through the various textures of the body. But, be this as it may, there is no doubt but that the blood has been too much overlooked in our pathological investigations; and it is to be hoped that the interesting chapter, devoted to this topic by Andral, will have a tendency to awaken the attention of the profession, especially that portion of it who reside in the Mississippi Valley, to the great importance of the subject. S. D. G.

